# Recombinant Forms of *Leishmania amazonensis* Excreted/Secreted Promastigote Surface Antigen (PSA) Induce Protective Immune Responses in Dogs

**DOI:** 10.1371/journal.pntd.0004614

**Published:** 2016-05-25

**Authors:** Elodie Petitdidier, Julie Pagniez, Gérard Papierok, Philippe Vincendeau, Jean-Loup Lemesre, Rachel Bras-Gonçalves

**Affiliations:** 1 IRD, UMR 177 INTERTRYP IRD CIRAD, Montpellier, France; 2 Virbac Animal Health, Carros, France; 3 University Hospital of Bordeaux, Laboratoire de Parasitologie-Mycologie, Bordeaux, France; 4 Université de Bordeaux, UMR 177 INTERTRYP IRD CIRAD, Bordeaux, France; Institut Pasteur de Tunis, TUNISIA

## Abstract

Preventive vaccination is a highly promising strategy for interrupting leishmaniasis transmission that can, additionally, contribute to elimination. A vaccine formulation based on naturally excreted secreted (ES) antigens was prepared from *L*. *infantum* promastigote culture supernatant. This vaccine achieved successful results in Phase III trials and was licensed and marketed as CaniLeish. We recently showed that newly identified ES promastigote surface antigen (PSA), from both viable promastigotes and axenically-grown amastigotes, represented the major constituent and the highly immunogenic antigen of *L*. *infantum* and *L*. *amazonensi*s ES products. We report here that three immunizations with either the recombinant ES *La*PSA-38S (rPSA) or its carboxy terminal part *La*PSA-12S (Cter-rPSA), combined with QA-21 as adjuvant, confer high levels of protection in naive *L*. *infantum-*infected Beagle dogs, as checked by bone marrow parasite absence in respectively 78.8% and 80% of vaccinated dogs at 6 months post-challenge. The parasite burden in infected vaccinated dogs was significantly reduced compared to placebo group, as measured by q-PCR. Moreover, our results reveal humoral and cellular immune response clear-cut differences between vaccinated and control dogs. An early increase in specific IgG2 antibodies was observed in rPSA/QA-21- and Cter-rPSA/QA-21-immunized dogs only. They were found functionally active *in vitro* and were highly correlated with vaccine protection. In vaccinated protected dogs, IFN-γ and NO productions, as well as anti-leishmanial macrophage activity, were increased. These data strongly suggest that ES PSA or its carboxy-terminal part, in recombinant forms, induce protection in a canine model of zoonotic visceral leishmaniasis by inducing a Th1-dominant immune response and an appropriate specific antibody response. These data suggest that they could be considered as important active components in vaccine candidates.

## Author Summary

Visceral leishmaniasis (VL), a potentially fatal disease caused by *L*. *infantum*, represents perfectly the need for a “One Health” approach for disease control, since it affects both humans and dogs, with similar clinical outcome and T-cell mediated immunity commitment. The dog vaccine development is highly required as our present resources for VL treatment and control have a limited effectiveness. It would represent the most convenient and efficient control way to decrease the dog-sandfly-dog transmission cycle, essential for human incidence reduction. The results indicate that recombinant forms of soluble promastigote surface antigen (PSA) are very promising effective vaccine candidates against canine VL. The elicited immune responses effectively reduced parasite load in *in vitro* pre-infected macrophages and in experimentally infected dogs. Through this approach, we aim to reduce the number of infected animals developing progressive infections thereby positively influencing human public health.

## Introduction

Leishmaniasis is among the most severe parasitic infections affecting humans and dogs in the world. It is the second-highest number of deaths caused by parasites worldwide. Leishmaniasis is remarkably associated with poverty and is an important part of neglected and uncontrolled tropical diseases [1]. Infection is provoked by protozoans of the genus *Leishmania*, transmitted by the bite of different species of phlebotomine sandflies. They replicate within host mononuclear phagocytes [2–4]. In humans, *Leishmania* parasites cause a wide spectrum of human diseases ranging from asymptomatic disease, self-healing cutaneous (CL), to disfiguring diffuse cutaneous leishmaniasis (DCL) or mutilating mucosal infections (MCL), and from subclinical to acute visceral disease (VL) that results in death in susceptible people, causing more than 59,000 deaths annually [1,5].

Anthroponotic visceral leishmaniasis in India and in Central Africa is caused by *L*. *donovani* and is characterized by the absence of animal reservoirs, making man the disease reservoir. Canids (wild and domestic) are unequivocally recognized as the main reservoir, continuously supplying the transmission cycle of *Leishmania infantum* in Old World *and L*. *chagasi* (its synonym in the New World). Zoonotic VL is found in the Mediterranean region, several Middle East, African and Asian countries, in South and Central America and probably in southern US [6–8]. VL increased incidence and severity are linked to parasite reservoir migrations, new insect vector localization due to environmental changes, co-infection with immunosuppressive diseases, disease urbanization, deforestation, and poverty [9–13]. In southwestern Europe, at least 2.5 million dogs are probably infected [14] and involved in the transmission cycle of *L*. *infantum* in humans. In the absence of effective and low-cost drugs for mass administration, priority control measures for elimination aim at reducing the transmission of VL. They include clinical, serological and parasitological diagnosis, treatment by chemotherapy, reduction of the vector population and preventive infectious sandfly bites by using several topical insecticides [15]. These actions are difficult to improve, expensive and weakly effective. Therefore, preventive canine vaccination is a highly promising strategy for interrupting VL transmission and could contribute to elimination [11,16]. Furthermore, vaccine development is a promising perspective for human leishmaniasis, as a successful chemotherapy restores impaired cellular immune responses [17] and individuals recovering from leishmaniasis are usually protected against a further infection [18–21]. The elaboration of a safe, efficient and inexpensive anti-leishmanial vaccine provides a substantial goal for global public health for animals and humans.

Our interest in *Leishmania* ES molecules was supported by previous studies showing their immunological properties on macrophage functions and in protection against challenge [22–29]. In recent years, a vaccine based on *L*. *infantum* promastigote ES (*Li*ESAp) achieved successful results in Phase III trials [23,28]. This vaccine was commercialized in Europe as CaniLeish since 2011 [23,28,30]. A prototype of this vaccine (*Li*ESAp/MDP) conferred 93% protection under field condition in France [28]. CaniLeish (LiESAp/QA-21) vaccine provided a significant reduction in the risk of progressing to active infection or overt disease with a clinical efficacy of 68% [30] in a context of a cumulative incidence of 80% of *Leishmania* infection [31]. More recently, we identified and characterized *La*PSA-38S and *Li*PSA-50S as major immunodominant ES components of *L*. *amazonensis* and *L*. *infantum* promastigotes, respectively [32]. These proteins were selectively recognized by vaccinated and protected dogs and human cells from immune individuals [24,32]. Members of this multi-gene family and the existence of membrane-bound members of the PSA family, such as the *PSA-2* gene, have been well documented [33–37]. *Leishmania* PSA proteins are involved in resistance to complement lysis and in macrophage adhesion/invasion *via* the complement receptor 3 (CR3) [38,39]. PSA-2 complex proteins induce a potent Th1 response in humans [40], and confer protection against a virulent challenge in mice [41]. In *L*. *infantum* and *L*. *major*-protected humans, we also clearly demonstrated that the native ES *La*PSA-38S protein in recombinant form (rPSA) evidenced a Th1-dominant response within an overall mixed Th1/Th2 profile and a cytotoxic response [24]. We hypothesize that this molecule might represent a potential candidate for a next second generation vaccine design.

Current recombinant technology leading to a highly purified/defined vaccine formulation increased vaccine safety, stability and reproducibility by decreasing batch to batch variation, compared to the already licensed first generation vaccines. This strategy should decrease the risk of adverse reactions associated with complex vaccine. Moreover, administering just the most immunogenic protein produces a more targeted immune response focused on identified protective antigens. We report here a vaccination trial on naive dogs using the recombinant ES *La*PSA-38S antigen (rPSA) expressed in *L*. *tarentolae* cultured in defined serum-free medium or its carboxy terminal part (Cter-rPSA) expressed in *E*. *coli*. Recombinant *La*PSA-12S antigen (Cter-rPSA) is a truncated polypeptide corresponding to the protease-resistant immunodominant carboxyl-terminal domain of the protein [42]. In this first investigation using these recombinant proteins and including a limited number of animals in a short term survey, dogs were randomly included into three experimental groups that received three subcutaneous injections at 4-week interval of either buffer saline or adjuvanted rPSA or adjuvanted Cter-rPSA. Vaccinated dogs had significantly higher levels of specific and efficient IgG2 antibodies and developed an early and specific Th1-dominant cellular immune response (increased NO-mediated anti-leishmanial macrophage activity in response to higher levels of IFN-γ). This dominant Th1 immune profile was found to be correlated with a good protection against an intravenous *L*. *infantum* promastigote challenge.

## Results

### Clinical evolution of dogs

No local and/or general adverse reactions, no hyperthermia, no body weight loss were seen in all immunized dogs upon vaccination. The candidate vaccine tolerance was satisfactory.

Immunized dogs were checked monthly for the appearance of external clinical manifestations until 6 months after administration of the parasites. No obvious clinical signs of leishmaniasis were noted in any of the dogs from the vaccinated and placebo groups until 6 months after challenge.

### Parasitological evolution of dogs

**Fig 1:**
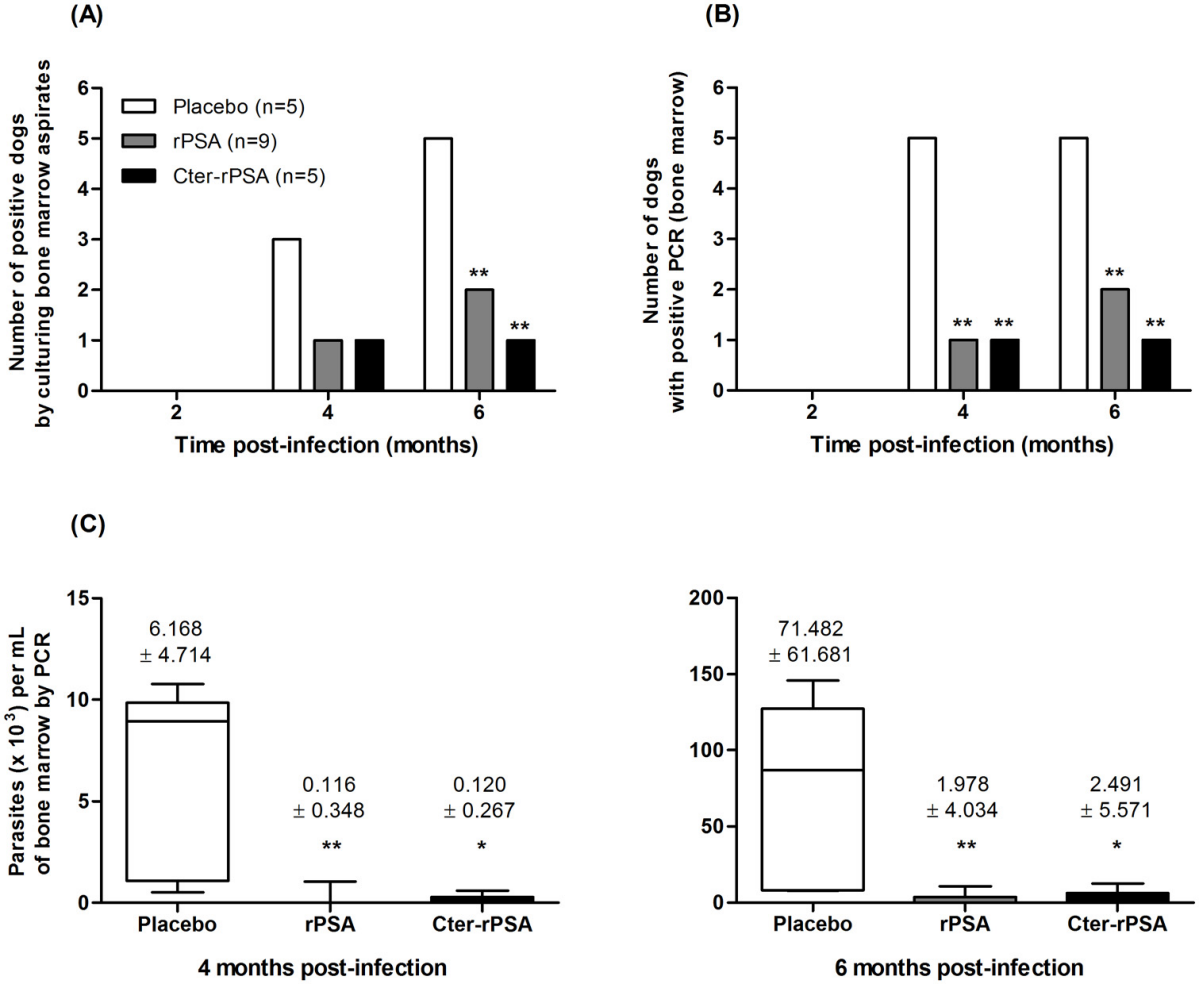
Parasitological evaluation of placebo and vaccinated dogs. The presence of (A) live *Leishmania* parasites was highlighted by subculture analysis of bone marrow aspirates isolated from dogs of placebo (n = 5), rPSA/QA-21 (n = 9) and Cter-rPSA (n = 5) groups at 2, 4 and 6 months post-challenge (PC). A sample was considered as positive when *Leishmania* parasites were detected during the seeding or subculture analysis. The presence of (B) *Leishmania* DNA and (C) the parasite load in bone marrow aspirates of dogs of each group were assessed by quantitative PCR. Dogs were considered as positive when the titer was superior to 40 parasites per mL. Data are expressed as (B) the number of dogs with positive PCR at each time points post-challenge and (C) the mean number of parasites per mL of bone marrow aspirates at different times post-challenge (4 and 6 months) (* *p*<0.05, ** *p*<0.01, *** *p*<0.001).

The occurrence of parasites in sub-cultures (Fig 1A), the presence of parasite DNA (Fig 1B) and the parasite loads using q-PCR (Fig 1C) were determined in bone marrow aspirates for the three groups of dogs at 2, 4 and 6 months post-challenge. A major difference in the number of infected animals between placebo group and the groups of vaccinated dogs during the post-challenge period was noted (Fig 1). In the control group (n = 5), three and five dogs were culture positive at 4 months and 6 months respectively, whereas all the animals were PCR-positive at every time point. By contrast, only two (culture-positive) and one (PCR-positive) out of five dogs vaccinated with Cter-rPSA were found parasite-positive at 4 months and only one remained infected (PCR- and culture- positive) at 6 months. Similarly, one (11.1%) and two (22.2%) out of 9 dogs vaccinated with rPSA were found culture- and PCR- positive at 4 months and 6 months, respectively. Parasite loads are shown in Fig 1C. Mean values were significantly lower in vaccinated dogs (rPSA: *p* = 0.019 and 0.004; Cter-rPSA: *p* = 0.018 and 0.031) than in placebo group at 4 and 6 months post-infection, respectively.

### Humoral immune response

**Fig 2:**
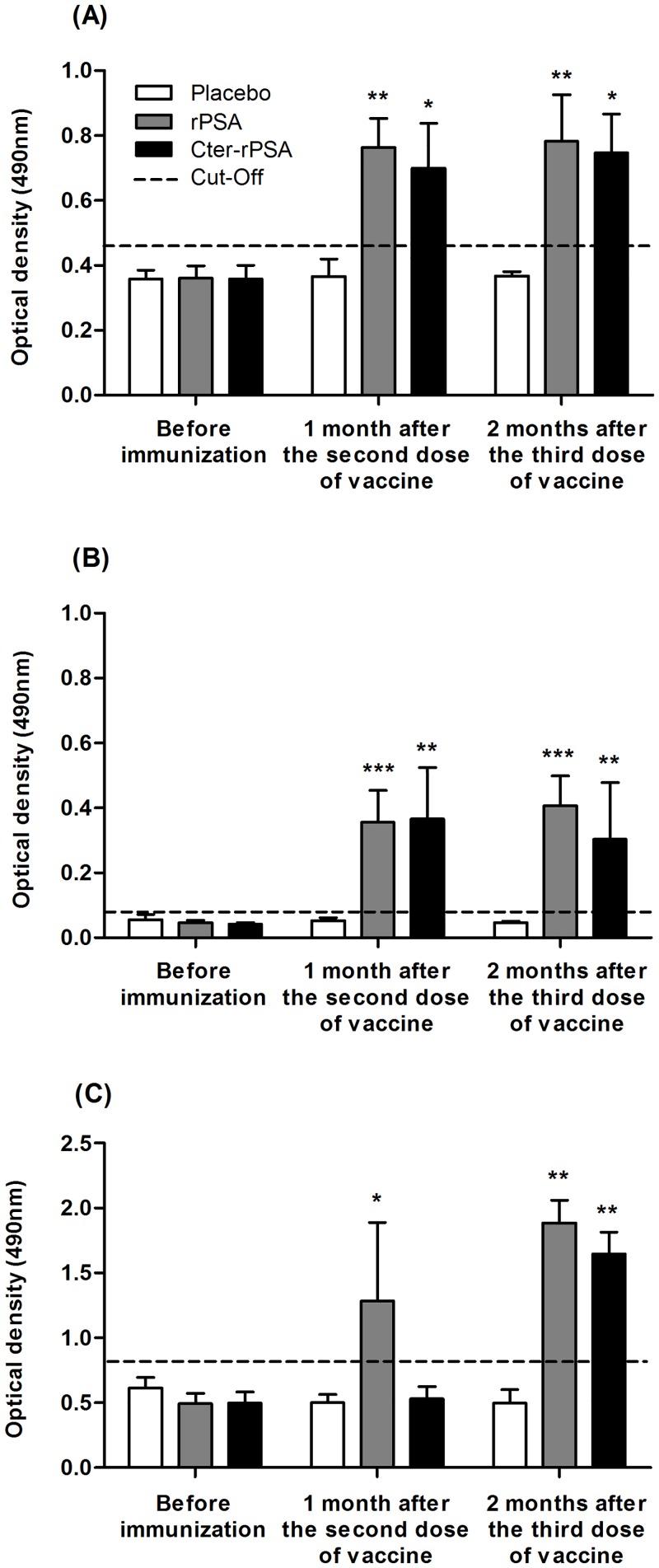
Vaccine specific serological responses as detected by Enzyme-Like Immunosorbent Assay (ELISA). Evolution of levels of (A) anti-rPSA, (B) anti-Cter-rPSA and (C) anti-*Li*ESAp specific IgG2 antibodies was assessed in serum samples isolated from dogs of each group immediately before immunization and at different times post-immunization: first month after the second dose and two months after the third dose. Each serum sample was tested in triplicates. Cut-off value was calculated using the following formula: mean OD in sera from all dogs before immunization + 3 standard deviations. Values represent means OD +/- standard deviation of triplicate experiments (* *p*< 0.05, ** *p*<0.01, *** *p*<0.001).

As shown in Fig 2, specific IgG2 antibody responses against rPSA (A), Cter-PSA (B) and *Li*ESAp (C) were assessed by ELISA in all serum samples. A weak specific IgG2 response was measured in all dogs immediately before immunization and in dogs from the placebo group at different times post-immunization: 1 month after the second dose of vaccine and 2 months after the third dose. Dogs vaccinated with rPSA/QA-21 and Cter-rPSA/QA-21 had significantly higher levels of anti-*L*iESAp [except for Cter-rPSA group: *p* = 0.0420 and *p* = 0.4206, respectively (Fig 2C)], anti-rPSA [*p* = 0.0033 and *p* = 0.0010, respectively (Fig 2A)] and anti-Cter-rPSA [*p* = 0.0119 and *p* = 0.0079, respectively (Fig 2B)] antibodies. IgG2 antibodies were detected as early as one month after the second vaccine candidate injection compared to placebo group for the same period. Specific IgG2 antibody titers remained significantly higher in vaccinated dogs compared to those of placebo animals two months after the third injection. Overall, IgG2 anti-leishmanial response was significantly higher in vaccinated groups compared to placebo group.

**Fig 3:**
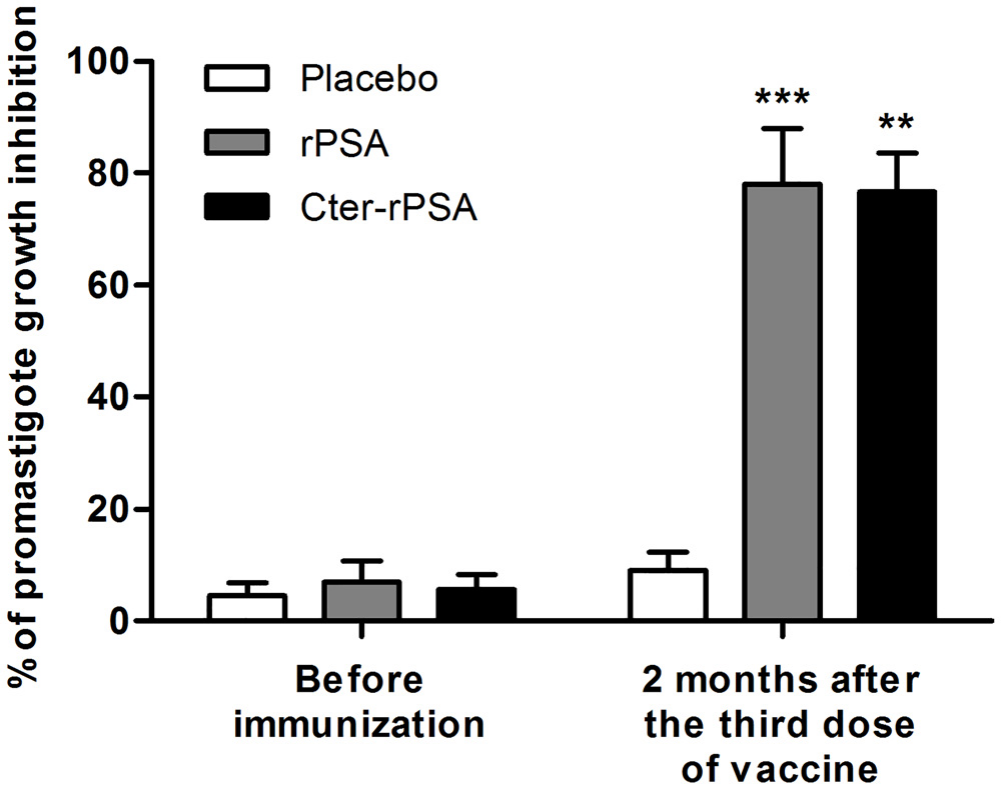
Effect of immunized dog sera on the proliferation of *L*. *infantum* promastigotes. The anti-proliferative effects were assessed on the growth of *L*. *infantum* promastigotes previously exposed for 30 min to serum samples from placebo (n = 5) and vaccinated dogs [rPSA (n = 9) or Cter-rPSA (n = 5)], collected before immunization and at 2 months post-vaccination, and then washed and cultured for 3 days at 25°C in 5 mL of RPMI medium supplemented with 20% foetal calf serum (FCS). At day 3, parasites were counted by flow cytometry (FACSCanto, Becton Dickinson) to assess cellular viability and parasite concentration. Results are expressed in percentage of promastigote growth inhibition +/- standard deviation (* *p*<0.05, ** *p*<0.01, *** *p*<0.001).

Viability of *L*. *infantum* promastigotes untreated or treated for 30 min with placebo dog sera collected two months after the third injection was nearly identical. More than 90% of promastigotes remained healthy and viable (data in S1 Fig). In contrast, a 30 min exposure of promastigotes with vaccinated dog sera at the same time point revealed around 45% of viability. The anti-proliferative effects on *L*. *infantum* promastigote growth were evaluated by exposing parasites 30 min to serum samples from placebo and vaccinated dogs [rPSA (n = 9) or Cter-rPSA (n = 5)], washing and culturing for 72 h under standard culture conditions. As shown in Fig 3, promastigote treatment with placebo dog sera did not affect parasite’s growth during the whole culture time. However, an inhibitory effect was evidenced on the growth of promastigotes previously incubated with vaccinated dog sera collected two months after the third injection (rPSA: *p* = 0.0010, Cter-rPSA: *p* = 0.0079, respectively).

### Macrophage anti-leishmanial activity

**Fig 4:**
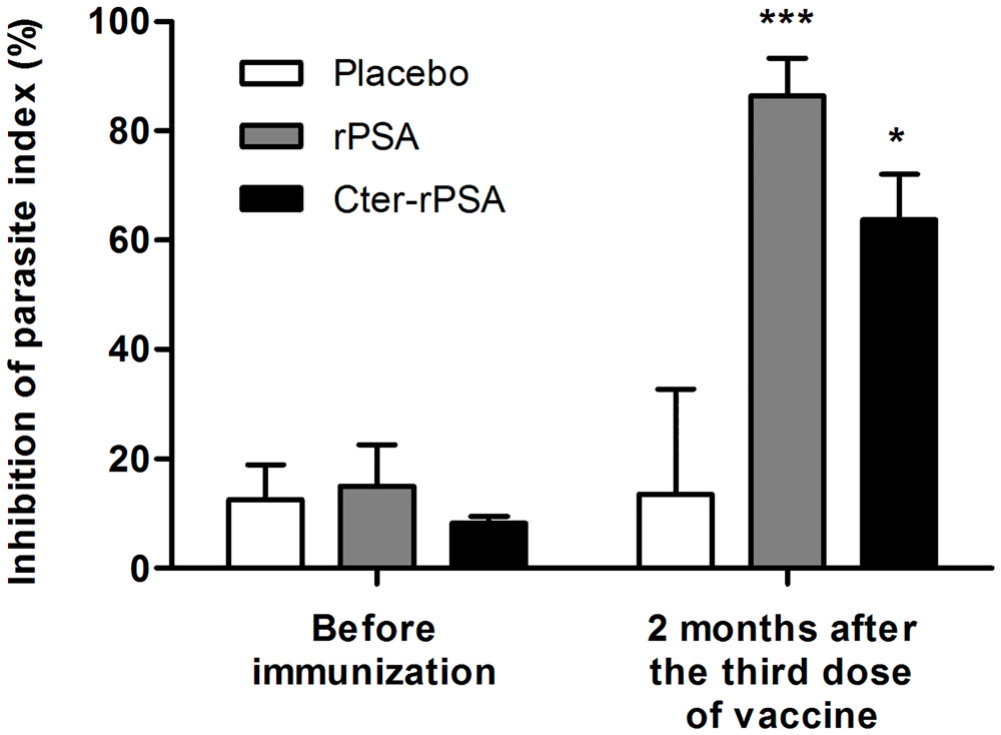
Anti-leishmanial activity of canine monocyte-derived macrophages in non-immune and immune dogs. The ability of pre-infected canine monocyte-derived macrophages to kill *Leishmania* parasites when they were exposed to autologous peripheral lymphocytes derived from PBMC was expressed as the percentage of parasitic index inhibition after *in vitro* infection with *Leishmania infantum* promastigotes (MHOM/MA/67/ITMAP-263) and 72 h incubation with and without autologous lymphocytes. Anti-leishmanial activity of co-cultured canine macrophages was evaluated immediately before immunization and two months after the third dose. Values represent means +/- standard deviation of duplicate experiments (* *p*< 0.05, ** *p*<0.01, *** *p*<0.001).

Macrophage ability to kill *L*. *infantum* when pre-infected macrophages were cultured in presence of autologous lymphocytes, expressed as percentage of parasite inhibition index, is presented in Fig 4. Anti-leishmanial activities were evaluated immediately before immunization and two months after the third injection. At starting point, the assay did not reveal any significant anti-leishmanial activity in any of the dogs, either placebo or vaccinated groups (Fig 4). By contrast, statistical differences were obtained between vaccinated and placebo groups two months after the completion of vaccine administration. As shown in Fig 4, higher anti-leishmanial activities were evidenced by infected macrophages from dogs vaccinated with rPSA/QA-21 or Cter-rPSA/QA-21 after co-culture with autologous lymphocytes, as demonstrated by a significant inhibition (86.4% (*p* = 0.0033) and 63.8% (*p* = 0.0119), respectively) compared to placebo group (13,5%).

**Fig 5:**
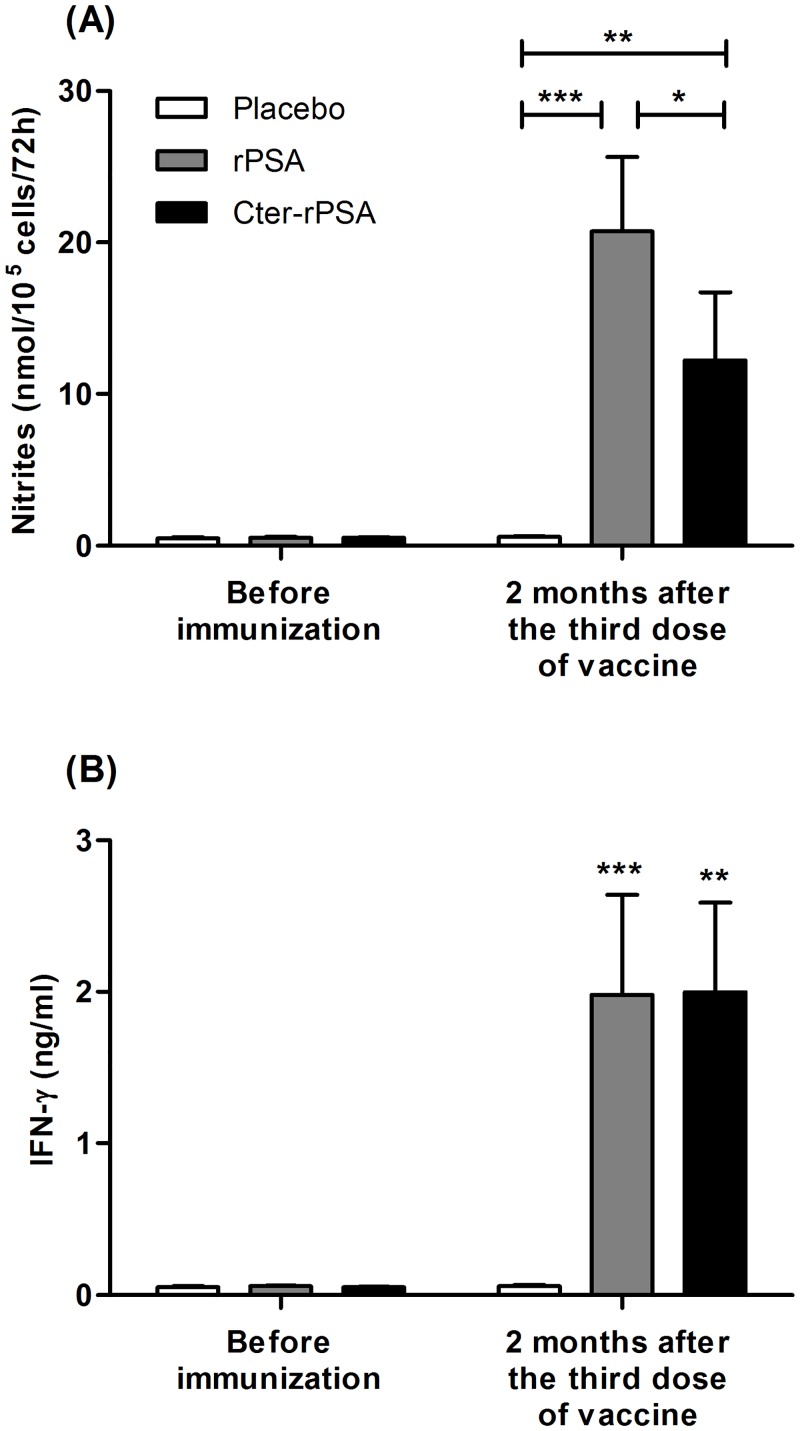
NO derivative and IFN-γ productions by 72 hours co-cultured canine macrophages from placebo and vaccinated groups of dogs (rPSA and Cter-rPSA). (A) NO_3_^-^/NO_2_^-^ accumulation in the same samples was used as an indicator of NO production by activated macrophages and was assayed using the modified Griess reaction according to Pinelli *et al*. [64]. (B) IFN-γ levels were determined by a two-site sandwich ELISA in cell culture supernatants of 72 h co-cultured cells. Values represent means +/- standard deviation of triplicate experiments (* *p*< 0.05, ** *p*<0.01, *** *p*<0.001).

NO_3_^-^/NO_2_^-^(NO derivatives) measurements in supernatants of co-cultured cells were directly correlated with NO pathway activation and parasite intracellular killing (Fig 5A). NO production in supernatants of all co-cultures was measured prior to immunization and two months after the third injection. Placebo dogs-infected macrophages synthesized low NO levels (around 0.6 nmol/10^5^ cells/72 hours) at the different time points analyzed prior to immunization and two months after the third injection. As shown in Fig 5A, the NO levels produced by macrophages from vaccinated dogs (rPSA/QA-21 and Cter-rPSA/QA-21) were significantly higher (20.75 +/- 4.88 nmol/10^5^ cells/72 hours, *p* = 0.0009 and 12.24 +/- 4.48 nmol/10^5^ cells/72 hours, *p* = 0.0069, respectively) than those evaluated before vaccination (0.52 +/- 0.06 nmol/10^5^ cells/72 hours) and in cell supernatants from placebo dogs 2 months after the vaccine course (0.50 +/- 0.07 nmol/10^5^ cells/72 hours). Moreover, the mean value was significantly increased in the group of dogs vaccinated with rPSA/QA-21 compared to the group of dogs vaccinated with Cter-rPSA/QA-21 (*p* = 0.012).

### Cytokine measurements

Cytokine contents in culture supernatants from placebo and vaccinated dogs were assessed prior to immunization and at two months after the third injection. As indicated in Fig 5B, prior to immunization, cell culture supernatants from all dogs expressed low IFN-γ levels (around 0.05 ng per mL). No increase in IFN-γ level was measured in supernatants of co-cultured pre-infected macrophages from placebo group two months after the third injection. By contrast, in supernatants of pre-infected macrophages co-cultured with autologous lymphocytes from rPSA/QA-21- or Cter-rPSA/QA-21-vaccinated dogs, significant higher IFN-γ levels were measured (1.98 +/- 0.66 ng/mL, *p* = 0.0010 and 1.99 +/- 0.59 ng/mL, *p* = 0.0079, respectively) than in those from placebo (0.060 +/- 0.007) or pre-immune dogs (0.052 +/- 0.006) (Fig 5B).

IL-4 and IL-10 levels in co-culture supernatants from all groups were low and not significantly different prior to immunization, and 2 months after completion of the vaccination protocol (data in S2 Fig).

## Discussion

In this study, we report that naive dogs immunized thrice with either the recombinant ES *La*PSA-38S (rPSA) or its recombinant C terminal part *La*PSA-12S, (Cter-rPSA), and combined with QA-21 as adjuvant, conferred a marked protection against *L*. *infantum* promastigote infection. A negative bone marrow parasite load was evidenced in 77.8% and 80.0% vaccinated dogs respectively at 6 months post-challenge follow-ups. The parasite burden was significantly reduced in three infected vaccinated dogs, but the short term survey did not allow the observation of any delayed disease progression or a role as reservoirs.

Investigations on potential *Leishmania* antigen screening and best adjuvant for vaccines have required substantial effort [11,43]. First vaccine attempts based on killed or attenuated parasites, conceptually simple to produce in endemic areas at low cost, gave disappointing results in field trials [9,44]. Second-generation vaccines are now available. Only two dog vaccines achieved successful results in Phase III trials: 1- Leishmune (Fort Dodge Animal Health), based on a glycoproteic complex (fucose mannose ligand) from *L*. *donovani* adjuvanted with QS-21 and deacylated saponins of *Quillaja saponaria*, licensed and used in Brazil [11,45,46] and 2- CaniLeish (Virbac Animal Health) a formulation related to the *Li*ESAp vaccine using *L*. *infantum* promastigote ES antigens from culture supernatant combined to QA-21 as adjuvant [22,25,27,28,47,48], licensed and marketed in Europe since 2011. A primary vaccination course of three injections at three week intervals, followed by annual booster vaccinations, is required with CaniLeish. The same protocol including two booster injections was scheduled in this study, which represent an important drawback in endemic areas. Various *Leishmania* molecules have already been reported as promising vaccine candidates (reviewed by [49,50]), however very few recombinant vaccines have succeeded beyond the laboratory rodent stage. This in part is linked to laboratory animal models which do not mimic natural infection. Only LeishTec vaccine (recombinant A2 antigen plus adjuvant) led partial protection (40%) versus *L*. *chagasi* promastigote challenge and has been marketed as a canine vaccine (Hertape Calier) in Brazil. Other vaccine candidates, such as the multicomponent Leish-111f fusion protein including TSA, LmSTI1, and LeIF antigens [31,51], gave disappointing results in dogs. Similarly, trials with recombinant cysteine peptidases plus canine IL-12, or recombinant Histone H1, hydrophilic acylated protein B1, or *Leishmania* activated C kinase receptor (LACK) analog, did not protect dogs from *Leishmania* infection [52]. We report here a successful vaccination study with recombinant *Leishmania* proteins in dogs. Efficacy of vaccination was corroborated by both qPCR and culture methods and clear differences in humoral and cellular immunity were evidenced between placebo and vaccinated groups.

Our results demonstrated clear-cut immune response differences between vaccinated and placebo dogs. An early increase in specific IgG2 antibodies, correlated with protection, was only evidenced in rPSA/QA-21- and Cter-rPSA/QA-21-immunized dogs. The IgG1 antibodies are associated with susceptibility, disease severity and correlates with a Th2 response [53–55], while IgG2 are predominant in naturally resistant or vaccinated dogs and are associated with an appropriate Th1-dominant response [56–58]. More recently, we provided direct evidence that cooperation of both humoral and cellular immune response might be essential for protection in *Li*ESAp/muramyl dipeptides immunized dogs [22]. We demonstrated here that inactivated sera from rPSA/QA-21- or Cter-rPSA/QA-21-vaccinated dogs supported promastigote killing and a significant parasite growth culture inhibition. Our data evidenced that an appropriate antibody response, such as mediated by anti-rPSA and anti-Cter-rPSA antibodies, might play a major role in canine VL protection.

Recent technological advances have allowed a global analysis of *Leishmania* secretome [59,60]. However, secretome functions are poorly known since few of these molecules have been extensively characterized [59,61]. Analysis of recombinant rPSA and Cter-rPSA amino acid sequences reveal that Cter part contains a Threonine/Serine-rich domain and Proline/Cysteine-rich regions. These regions are relatively conserved between *Leishmania* species but their lengths vary considerably and they can be totally absent in some PSAs [33]. The immunodominant humoral and cellular responses to this region might be linked to its proteolytic stability and hydrophobicity [36,42,62]. This suggests that ES PSA might have an important immunoregulatory role between parasites and their target cells and on the immune responses. Promastigotes might secrete PSA to modify macrophage functions even before parasite engulfment. Analogously, amastigotes secrete PSA, which might be transported across the parasitophorous vacuole membrane, to interfere with macrophage signaling pathways, thereby preventing macrophage activation. So, future candidates might exist in *Leishmania* ES proteins and be used for new generation vaccine design.

Intracellular *Leishmania* parasite killing by macrophages is essential for cure. We previously developed an *ex vivo* canine macrophage-autologous lymphocyte co*-*culture system to investigate protective cellular response. This co-culture system was used to analyze NO pathway involved in macrophage parasite killing. Increased IFN-γ and NO production, as well as anti-leishmanial macrophage activity, were verified only in vaccinated dogs, and lasted two months after the full vaccination course. This further argues that *Leishmania* killing is mediated *via* an L-arginine NO pathway, induced by Th1 cytokines, mainly IFN-γ. All these points support the hypothesis that ES PSA and its carboxy terminal part, in recombinant forms, induce protection in canine VL by inducing a Th1-dominant immune response and an appropriate specific antibody response. In addition, the nature and magnitude of immune responses revealed by PSA formulations is very similar to those induced by the different *Li*ESAp formulations. This suggests that ES PSA is one of the main active compounds of the already licensed CaniLeish. As NO and IFN-γ are also involved in human leishmaniasis [63], monitoring these parameters might represent markers of a protective response for recombinant PSA or its carboxy-terminal part-based vaccine development and for large scale field studies. Altogether, these results, obtained in this experiment with a limited number of animals and with a short term survey, deserve further investigations to evaluate these vaccine candidates against a severe disease for both humans and dogs in natural conditions of infection.

## Materials and Methods

### Production and purification of vaccine candidates: *La*PSA-38S (rPSA) and *La*PSA-12S (Cter-rPSA) recombinant proteins

#### Preparation of *La*PSA-38S (rPSA) recombinant protein

The *LaPSA-38S* gene (GenBank accession number: FJ974054, http://www.ncbi.nlm.nih.gov/genbank), was cloned and identified as previously described [32], and was amplified from pBluescript-SK vector by PCR with Phusion High-Fidelity DNA Polymerase (Finnzymes) using the following primers: forward primer, F-PSA-38S (5’-CCATGGCGCAGTGCGTGCGTCGG-3’) and reverse primer, R-PSA-38S (5’-GCGGCCGCGTGATGGTGATGGTGATGATCGTGGTTCGCCAG-3’), containing *Ncol* and *Notl* restriction sites in each 5' end (underlined). DNA amplification was performed according to the following protocol: 5 min hot-start at 95°C followed by 30 cycles of 95°C for 30s, 62°C for 30s and 72°C for 90s, and a final extension at 72°C for 10 min. The purified PCR product was cloned in pCR2.1-TOPO TA vector using TOPO TA cloning Kit (Invitrogen) according to the manufacturer’s procedures. The transformed TOP10 *E*. *coli* cells (Invitrogen) were screened for the presence of recombinant plasmid with the *LaPSA-38S* insert by gene-specific PCR and analyzed with *Ncol* and *Notl* restriction enzymes. Isolated positive clones were sequenced. The insert was removed by *Ncol* and *Notl* digestion and subcloned into the *Ncol* and *Notl* insertion site of *Leishmania* expression vector pF4X1.4sat1 allowing selection with the antibiotic Nourseothricin to create the recombinant pF4X1.4-*La*PSA-38S plasmid. The resulting construct, encoding a full sequence of *La*PSA-38S secreted protein, was fused to a C-terminus (His6)-tag. *L*. *tarentolae* promastigotes were successfully maintained in continuous culture by successive passages of 5 x 10^5^ flagellates/mL every week into 10 mL of completely defined CDM/LP medium free of serum, macromolecules, proteins and cell contaminants as previously described [29]. For stable integration of the expression cassette into the 18S ribosomal RNA (ssu) locus, 10 μg of pF4X1.4sat1 plasmid containing *LaPSA-38S* gene was digested by SwaI restriction enzyme. Transfections of *L*. *tarentolae* promastigotes were performed by electroporation in 2 mm cuvettes using a Gene Pulser II (Biorad), a single pulse (5–6 msec) with the settings 450 V and 450 mF. After transfection, cells were transferred into a fresh CDM/LP medium [29]. 100 μg/mL of Nourseothricin was added 24 h after electroporation to select stable transformants. One week later, only nourseothricin-resistant cells survived. Transgenic cells were selected as single colonies on the supplemented CDM/LP-agar medium containing 100 μg/mL Nourseothricin (Jena Bioscience, Germany) as selective antibiotic. Mass culture and protein purification were manufactured by Virbac Company in GMP conditions. Culture amplifications were performed on CDM/LP medium. The culture supernatant containing *L*. *amazonensis* ES *La*PSA-38S, released by the parasite during its growth, was recovered at the late stationary phase of growth. The only proteins in the medium, with their native conformation, are parasite-produced. Recombinant ES PSA was purified from concentrated culture supernatant through Ni-NTA affinity chromatography. This purified recombinant *La*PSA-38S protein migrated as a 45 kDa band in SDS–PAGE gel [24].

#### Preparation of recombinant *La*PSA-12S (Cter-rPSA) protein

The *LaPSA-12S* gene (GenBank accession number: FJ974053) is a truncated *LaPSA-38S* gene corresponding to the C-terminal part of *La*PSA-38S named Cter-rPSA, as previously described [32]. *La*PSA-12S is a polypeptide of 119 amino acids with a calculated molecular weight of 11.9 kDa. A pBluescript-SK vector with the cDNA encoding for *LaPSA-12S* gene was digested with specific endonucleases BamHI and KpnI (Eurogentec), compatible with multiple cloning site of pQE-31 vector (Qiagen). The final *Bam*HI–*KpnI*-generated DNA fragment was extracted from agarose gel and purified using Wizard DNA clean-up system (Promega) following the manufacturer’s procedures. The purified DNA fragment was used in a ligation reaction in the presence of the *Bam*HI–*KpnI*-digested and dephosphorylated pQE31 vector. The ligation product was transformed and propagated in *E*. *coli* M15 strain (Stratagene), purified and sequenced in both strands. The clones containing the correct coding frame were selected and used to prepare and purify the recombinant protein. Cter-rPSA was purified with Ni-NTA affinity chromatography. This purified truncated recombinant *La*PSA-12S protein migrated as a 16 kDa and 36 kDa bands in non-reduced SDS–PAGE gel. The band of 36 kDa corresponds to the 16 kDa dimer form (S3 Fig).

### Animals and study design

Nineteen young adult dogs, 10 males and 9 females, between 2 and 4 years old, were selected on clinical and serological criteria from a colony of naive Beagles from the kennel CEDS (Domaine des Souches, Mezilles, France). Dogs were housed at the animal facility of the National Veterinary School of Lyon (ENVL, France) in the “Unité d’Etudes PréCliniques” (UEPC) during the time course of the experiment under conditions designed to exclude any possible natural leishmanial infections. They were well-fed animals under constant scrutiny of health problems by a veterinarian and had all received their yearly routine vaccinations. Care and management of dogs were carried out according to ethical guidelines laid down in the National Veterinary School of Lyon (ENVL). Protocols were submitted to and approved by ethics committee of the ENVL (N° ICLB 135/08) and performed according to recommendations to limit the number of animals and long-term experimentations. All dogs had a specific code/ID throughout the experiment. The animals were maintained in quarantine for a period of 30 days before the initiation of the experiment. Prior to vaccination, blood was collected and then sera and genomic DNA of all dogs were separated and extracted in order to exclude any infected dog. Beagles were randomized by sex and age into three experimental groups and the study was performed in a double–blind randomized fashion. Dogs of each group received three subcutaneous injections at a 4-week interval of either freeze-dried dose of 1 mL buffer saline (Placebo group, n = 5), 25 μg recombinant *La*PSA-38S adjuvanted with 60 μg QA-21 (rPSA group, n = 9) or 25 μg recombinant *La*PSA-12S formulated with 60 μg QA-21 (Cter-rPSA group, n = 5). Two months post-immunization, all dogs were challenged by intravenous injection of 10^8^ infective promastigotes of *L*. *infantum* (MHOM/MA/67/ITMAP-263 strain, clone 2). Primary cultures of virulent promastigotes, differentiated from amastigotes isolated from the spleen of heavily infected mice (BALB/c), were used for the virulent challenge.

### Detection of specific IgG2 to rPSA, Cter-rPSA and *Li*ESAp by Enzyme-Linked Immunosorbent Assay (ELISA)

Specific IgG2 antibody responses against rPSA, Cter-rPSA and *Li*ESAp were measured in the serum samples of control and vaccinated dogs by a standard ELISA procedure. Briefly, sera from immune or control dogs were added in triplicate at 1/50 dilution in PBS containing 0.05% Tween-20 to 96-well plates previously coated with rPSA (0.1 μg per well, batch #A50054), Cter-rPSA (0.1 μg per well, batch #070425) or *Li*ESAp (1 μg per well, batch #0011). After 1 h incubation at 37°C, plates were washed extensively with PBS-0.05% Tween-20 and incubated for 30 min at 37°C with secondary antibody (horseradish peroxidase-conjugated sheep anti-dog IgG2, 1/5000). After three washes in PBS-0.05% Tween-20, plates were developed with OPD substrate (with H_2_O_2_ in citrate buffer) and absorbance was read using microplate reader at 492 nm wavelength. For analysis, a threshold of positivity was estimated by calculating a cut-off using the following formula: mean OD in sera collected from all dogs at the starting point (before immunization) + 3 standard deviations.

### Effect of sera from unvaccinated and vaccinated dogs on the proliferation of *Leishmania infantum* promastigotes

Promastigotes of *Leishmania infantum* were collected by centrifugation and washed three times in PBS. A total of 5x10^6^ parasites were incubated (or not) with 100 μL of complement heat-inactivated serum of dog for 30 min, at dilution ¼ in culture medium. These sera were collected from all dogs 2 months after vaccination. Cell’s viability was assessed by trypan blue staining, the parasites were washed three times in PBS, and then cultivated at 25°C in 5 mL of RPMI medium supplemented with 20% heat-inactivated foetal calf serum (FCS). Parasites were counted daily for three days by flow cytometry (FACSCanto, Becton Dickinson). Results are expressed in percentage of promastigote growth inhibition at day 3.

### Assessment of canine monocyte-derived macrophages anti-leishmanial activity

Peripheral Blood Mononuclear Cells (PBMC) were obtained from heparinized peripheral blood by density centrifugation through Ficoll-Hypaque (GE Healthcare Life Sciences). Canine monocyte-derived macrophages (CM-DM) and non-adherent cells (i.e. lymphocytes) were prepared by differential adherence of PBMC as previously described [25,27]. CM-DM separated from lymphocytes were cultured for 5 days at 37°C and 5% CO2 in RPMI 1640 medium (BioWhittaker), supplemented with 2 mM glutamine, 10% heat-inactivated FCS, 100 μg/mL streptomycin and 100 IU/mL penicillin. They were infected with stationary-phase promastigotes of *L*. *infantum* (MHOM/MA/67/ITMAP-263 strain, clone 2) at a parasite: macrophage ratio of 5:1 for 150 min in LabTek 16-well glass chamber slides. Non-internalised parasites were removed by gentle washing. The cells were checked to verify that greater than 40% were infected. Infected macrophages were then incubated alone or in the presence of autologous lymphocytes at a lymphocyte: macrophage ratio of 2:1. After a 72 h co-culture, supernatants were collected for further analyses and the lymphocytes were removed by gentle washings. Macrophages were fixed with methanol and stained with Giemsa in order to determine the parasitic index. For assessment of anti-leishmanial activity, the percentages of infected cells and the number of amastigotes per macrophage were estimated in duplicate experiments by microscopic examination of Giemsa-stained preparation and were used to calculate the parasite index (PI) inhibition. Percentage of PI inhibition = 100 –[(mean number of amastigotes per macrophage × percentage of infected macrophages when macrophages were incubated with autologous lymphocytes) / (mean number of amastigotes per macrophage × percentage of infected macrophages in untreated macrophages)] × 100.

### Cytokine and nitric oxide measurements

NO_3_^-^/NO_2_^-^ accumulation in supernatants from 72 h co-cultured cells (pre-infected macrophages exposed to autologous lymphocytes) was used as an indicator of NO production by activated macrophages and was assayed by the Griess reaction using the nitrate/nitrite colorimetric assay of Alexis biochemicals (Enzo Life Sciences, France). The Griess reagent was modified according to Pinelli et *al*. [64].

IFN-γ, IL-4 and IL-10 levels were determined as previously described [25] in cell culture supernatants by a two-site sandwich ELISA using specific anti-dog IFN-γ (2 μg/mL), anti-dog IL-4 (1 μg/mL) and anti-dog IL-10 (1 μg/mL) antibodies (R&D Systems, Minneapolis, USA), biotinylated anti-dog IFN-γ (100 ng/mL), anti-dog IL-4 (50 ng/mL) and anti-dog IL-10 (50 ng/mL) antibodies (R&D Systems, Minneapolis, USA) and streptavidin conjugated to horseradish peroxidase (1/200) (R&D Systems, Minneapolis, USA). Absorbance values were read at 490 nm wavelength in an automatic microplate reader (Wallac Victor21420 Multilabel counter, Perkin-Elmer life sciences). Standard curves for IFN-γ, IL-4 and IL-10, respectively, were performed by the use of recombinant canine proteins (R&D Systems, Minneapolis, USA).

### Clinical follow-up and assessment of parasite load

The health status of the animals was routinely followed by veterinarians (including appetite, physical examination and physical activity). The dogs were monitored for 3 weeks after each injection. Local tolerance was investigated by direct visual examination and any lesions were scored daily over a period of 14 days after each injection. General tolerance was investigated by means of a weekly general clinical examination and a daily general health evaluation with rectal temperature measurement. Body weights were measured once a week throughout the trial period.

Dogs were monitored for parasite establishment and subsequent development of the disease by routine screening for classical clinical signs and parasite isolation. Infection was assessed at 2, 4 and 6 months post-challenge. For that, dogs were anesthetized and bone marrow aspirates were collected by sternal puncture into citrate tubes to assess parasite load.

The presence of *Leishmania* parasites was determined by culturing parasites in NNN biphasic medium at 25°C. Bone marrow samples (about 500 μl) were cultured in NNN biphasic medium (containing 2 mL of RPMI-20% heat-inactivated FCS) for 1 week. Subcultures were weekly realized by adding 0.5 mL or 1 mL of culture sample in NNN medium containing 3 mL of RPMI-20% FCS (4 subcultures). The presence of parasites was determined by regular microscopic observation for 20 min in an inverted microscope at 400x magnification. When parasites were observed, the sample was considered as parasite positive.

The presence of *Leishmania* DNA was also assayed in bone marrow samples of all the enrolled dogs by real-time quantitative PCR (qPCR) as previously described for kinetoplast DNA amplification [65]. After lysis, the DNA of each bone marrow sample was extracted using a silica column (QIAamp DNA mini kit). The Stratagene (La Jolla, California, USA) MX 4000 system was used for amplification and detection. Optimization experiments led us to use the Stratagene qPCR master mix, 15 pmol of forward primer (CTTTTCTGGTCCTCCGGGTAGG), 15 pmol of reverse primer (CCACCCGGCCCTATTTTACACCAA), and 50 pmol of TaqMan probe (FAM-TTTTCGCAGAACGCCCCTACCCGC-TAMRA). Assays were performed with a 25 μL final volume with 1 μL of DNA sample. The standard curve was established from *Leishmania* DNA extracted from 5 × 10^6^ parasites; 1 μL of each serial dilution, ranging from 50,000 to 0.0001 parasites, was introduced into reaction tubes. TaqMan chemistry allowed two-step temperature (94 and 55°C) cycling over 45 cycles. Comparative quantification was performed by using a single copy gene, the DNA polymerase gene, as a normalizer. Primers and a probe described previously by Bretagne *et al*. [66] (TGTCGCTTGCAGACCAGATG [200 pmol], GCATCGCAGGTGTGAGCAC [200 pmol], and VIC-CCAGGCTCGAAGTTGTTGCTGCCC-TAMRA [200 pmol]) and the same working conditions as previously described for kinetoplast DNA amplification were used. Results were expressed as the number of parasites per mL of bone marrow aspirate. A sample was considered as positive when the established parasite concentration was superior to 40 parasites per mL.

### Statistical analysis

Data analysis was performed with GraphPad Prism version 5.03 for Windows, GraphPad Software (San Diego, California USA). Statistical significance of differences between groups was determined by Mann-Whitney-Wilcoxon test. A *p*-value ≤ 0.05 was considered significant.

### Ethics statement

The ENVL Ethical Committee approval confirms that this study was carried out in accordance with the G.R.I.C.E. “Ethical Committee Regulation applied to animal experimentation” guidelines (implemented in France in 2008) under project number 135.08.

## Supporting Information

S1 Fig Effect of immunized dog sera on the proliferation of *L*. *infantum* promastigotes. Promastigotes of *L*. *infantum* are exposed for 30 min to serum samples from placebo (n = 5) and vaccinated dogs [rPSA (n = 9) or Cter-rPSA (n = 5)], collected before immunization and 2 months post-vaccination. Cellular viability was assessed by flow cytometry (FACSCanto, Becton Dickinson) just after exposure. Values represent average percentage of promastigote viability +/- standard deviation (* *p*<0.05, ** *p*<0.01, *p*<0.001).
(TIF)

S2 Fig IL-4 and IL-10 productions by co-cultured cells from placebo and vaccinated (rPSA and Cter-rPSA) groups of dogs. (A) IL-4 and (B) IL-10 levels were determined by a two-site sandwich ELISA in cell culture supernatants of 72 h co-cultured cells. Values represent means +/- standard deviation of triplicate experiments.
(TIF)

S3 Fig Analysis of the production and purification of recombinant *La*PSA-12S protein by using Coomassie-stained SDS-PAGE gel. (lane 1) 5 μg of the purified recombinant *La*PSA-12S, (lane 2) 10 μg of the purified recombinant *La*PSA-12S, (lane 3) SeeBlue Pre-stained Standard. Staining by Coomassie blue reveals a band of about 16 kDa corresponding to the *La*PSA-12S protein in monomeric form and a band of about 36 kDa corresponding to the dimerized protein.
(TIF)
