# Supplementary figures and images for: Recombinant Forms of Leishmania amazonensis Excreted/Secreted Promastigote Surface Antigen (PSA) Induce Protective Immune Responses in Dogs
Source: PLoS Negl Trop Dis. 2016 May 25;10(5):e0004614. doi: 10.1371/journal.pntd.0004614 (PMC4880307; doi:10.1371/journal.pntd.0004614)

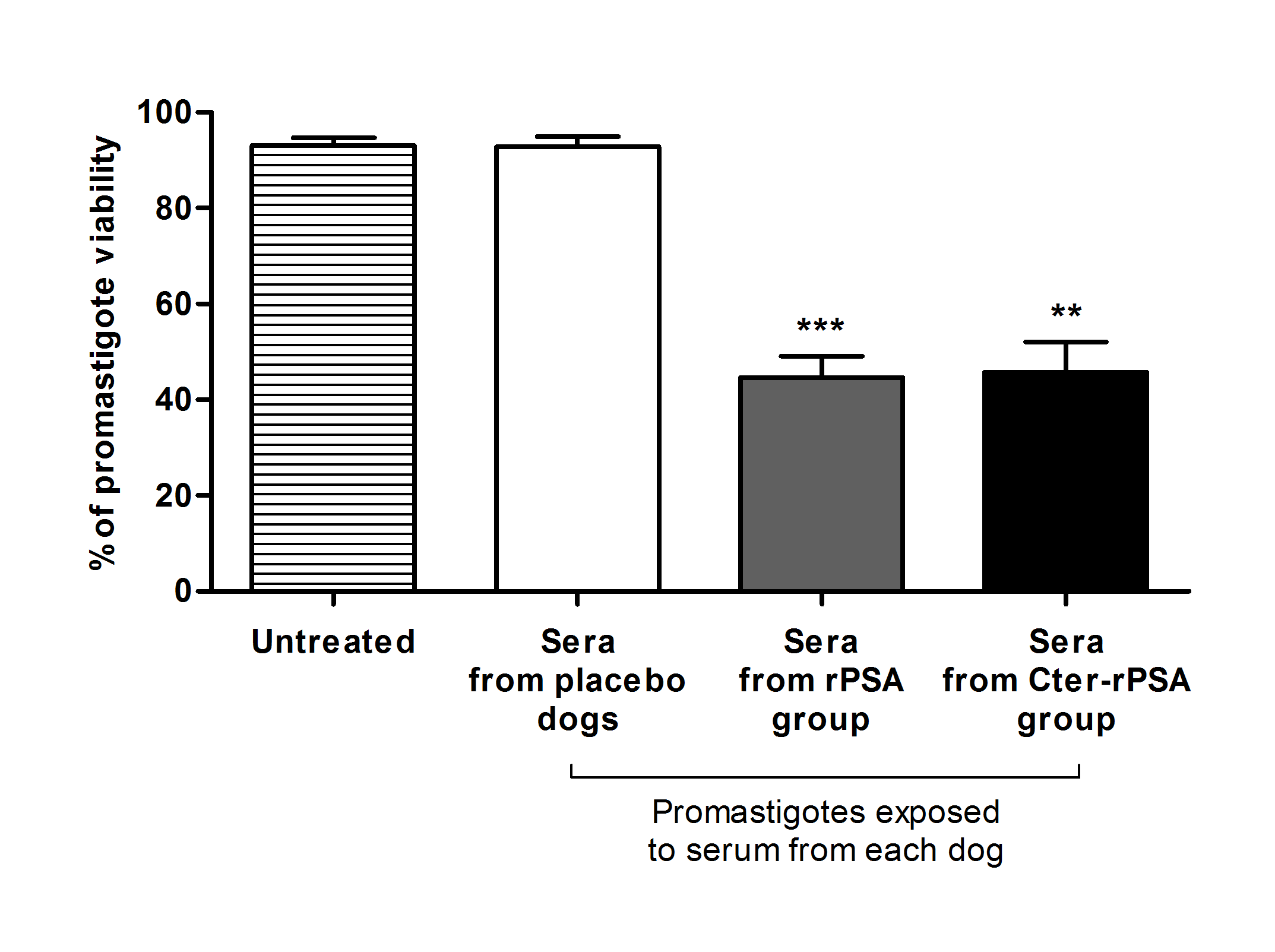

Supplement: S1 Fig — Promastigotes of L. infantum are exposed for 30 min to serum samples from placebo (n = 5) and vaccinated dogs [rPSA (n = 9) or Cter-rPSA (n = 5)], collected before immunization and 2 months post-vaccination. Cellular viability was assessed by flow cytometry (FACSCanto, Becton Dickinson) just after exposure. Values represent average percentage of promastigote viability +/- standard deviation (* p<0.05, ** p<0.01, p<0.001). (TIF) [file pntd.0004614.s001.tif]

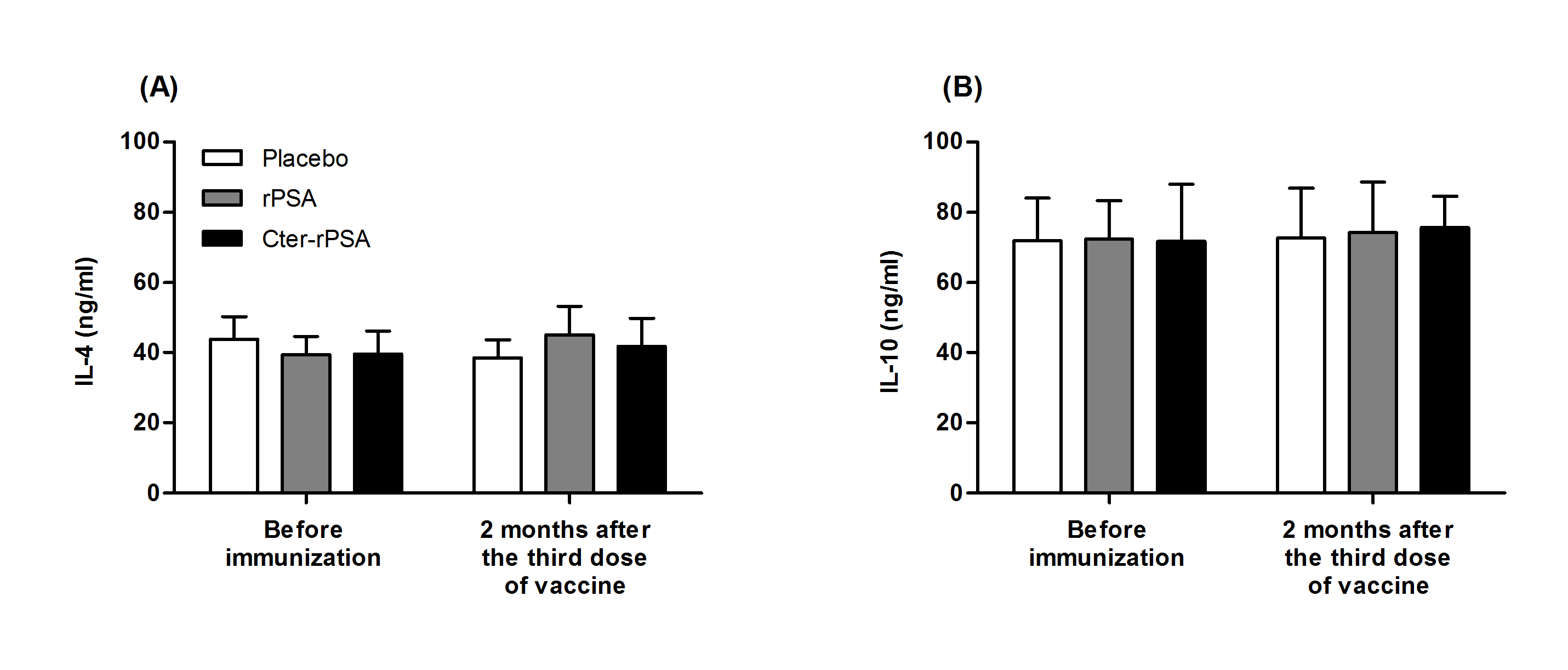

Supplement: S2 Fig — (A) IL-4 and (B) IL-10 levels were determined by a two-site sandwich ELISA in cell culture supernatants of 72 h co-cultured cells. Values represent means +/- standard deviation of triplicate experiments. (TIF) [file pntd.0004614.s002.tif]

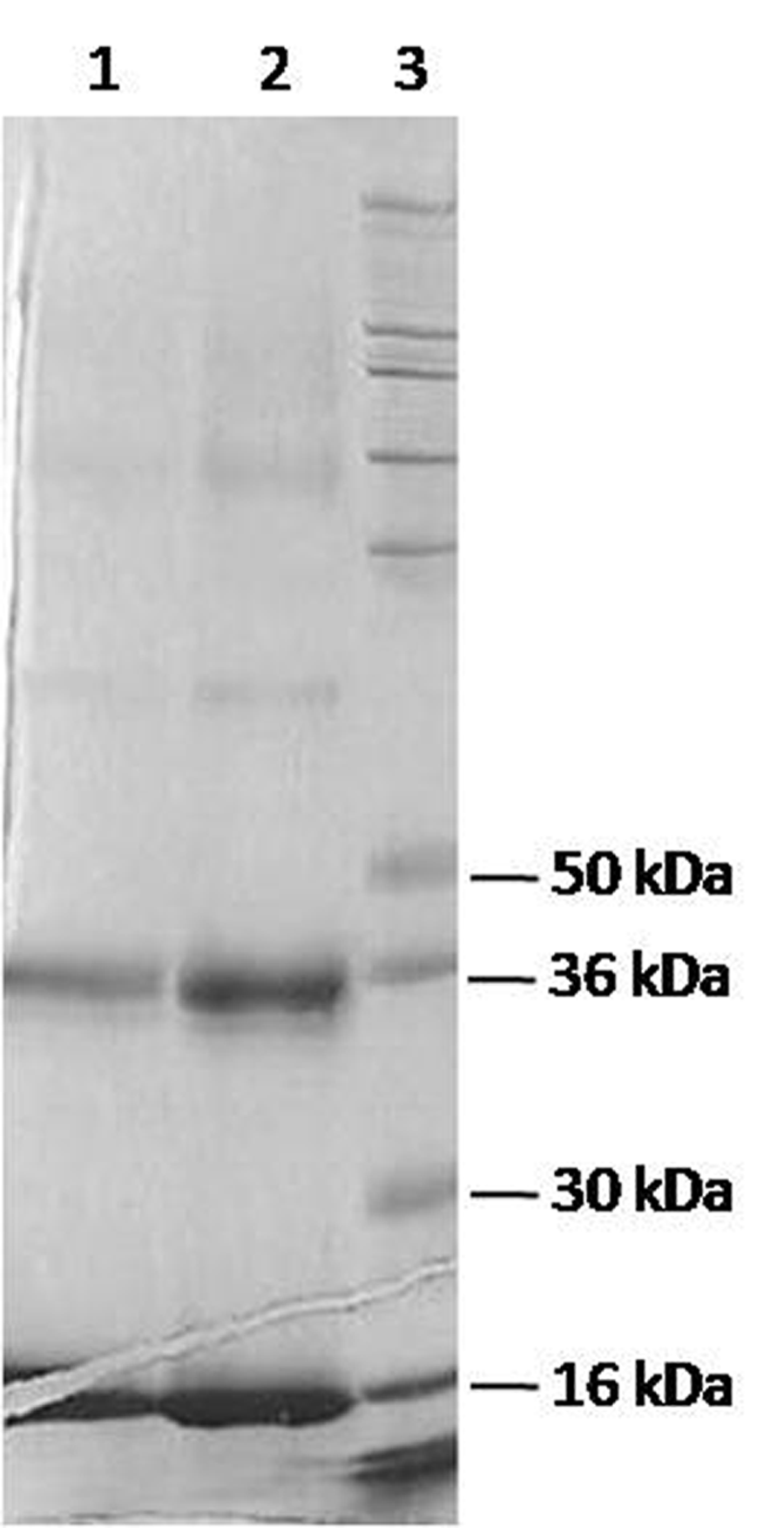

Supplement: S3 Fig — (lane 1) 5 μg of the purified recombinant LaPSA-12S, (lane 2) 10 μg of the purified recombinant LaPSA-12S, (lane 3) SeeBlue Pre-stained Standard. Staining by Coomassie blue reveals a band of about 16 kDa corresponding to the LaPSA-12S protein in monomeric form and a band of about 36 kDa corresponding to the dimerized protein. (TIF) [file pntd.0004614.s003.tif]
